# Belief in and use of traditional Chinese medicine in Shanghai older adults: a cross-sectional study

**DOI:** 10.1186/s12906-020-02910-x

**Published:** 2020-04-28

**Authors:** Benlu Xin, Siyu Mu, Teckkiang Tan, Anne Yeung, Danan Gu, Qiushi Feng

**Affiliations:** 1 Center for Quantitative Economics, Jilin University, Changchun, China; 2 Business School, Jilin University, Changchun, China; 3 Institute for Applied Learning Sciences and Educational Technology (ALSET), National University of Singapore, Singapore, Singapore; 4 Independent Researcher, New York City, NY USA; 5 Department of Sociology, National University of Singapore, AS1 04-30, 11 Arts Link, Singapore, 117570 Singapore

**Keywords:** Traditional medicine, Belief, Use, China, Shanghai, Older adults

## Abstract

**Background:**

Traditional medicine is broadly used across Asian societies for various medical conditions and health concerns. However, there remains a wide disparity between users and non-use, which makes it imperative to understand the factors affecting the lay perception and utilization of traditional medicine. This study systematically examined the demographic, medical, and socioeconomic factors affecting belief in and use of traditional medicine among older adults of Shanghai, China.

**Methods:**

We used the data from Survey of Life and Opinion on Shanghai Older Adults in 2013 with a sample of 3418 older adults aged 50 years or older. The multilevel logistic models were applied to examine the associations between faith and utilization of traditional medicine and a set of factors of the respondents, including demographics (gender, age, rural/urban residence), socioeconomic status (educational attainment, income, primary occupation), social support (marital status, social network), and disease/conditions. The associations between individual use of traditional medicine and the profile of socioeconomic development and the medical services conditions of local communities were also modelled.

**Results:**

We found that cardiovascular diseases, lung diseases, cancer, prostatitis, arthritis, and nervous system diseases were positive correlates for using traditional medicine. Older adults who had a cancer, a prostatitis, or a fracture had more faith in traditional treatment. Rural living, higher educational attainment, and white-collar occupation promoted the use of traditional medicine. A higher number of strong social ties and a tie connected with medical staff were positive factors of use as well.

**Conclusion:**

The belief in and use of traditional medicine were prevalent among older adults in Shanghai, China. Though not conclusive, our study suggested that traditional medicine in China appears to serve two distinct functions, namely complementary medicine for those socioeconomically advantaged whereas alternative medicine for those socioeconomically disadvantaged.

## Background

Traditional medicine is broadly used across Asian societies [1–5], not only applied for daily health maintenance, but also used in alternative treatments for cancers, strokes, and many other medical conditions. In some Asian societies such as China, Japan, and Korea, traditional medicine has been institutionalized to nearly or equivalent the degree as conventional medicine [6, 7]. Practitioners of traditional medicine are officially certified and regulated, and related therapy expenses are covered by the national health insurance schemes [8, 9].

Despite the institutional promotion of use of traditional medicine, there is evidence for a decline trend in using of traditional medicine in China among the general people from 1990 to 2004 [10]. At the same time, there continues to be considerable heterogeneity in perception and use of traditional medicine across and within Asian societies. A recent review [4], for example, reported that the prevalence of using traditional/alternative/complementary medicine ranged from 20.0 to 97.4% in the Southeast Asia countries. A study from Hong Kong [11] classified the attitude towards the use of traditional medicine into three types: holding belief in conventional medicine (63%), being sceptical on conventional medicine (24%), and holding faith on both traditional medicine and conventional medicine (14%).

In recent years, there is a growing body of literature on utilization of traditional medicine, in which researchers start to systematically understand health behaviors in regard to traditional medicine through the Knowledge, Attitude and Practice (KAP) model [12–14]. There is, however, limited understanding in the current literature of the factors influencing attitudes and use of traditional medicine in Asian countries. Demographic factors are not fully explored. Except for the most conclusive evidence that females are more likely to use traditional medicine than males [15–17], other results concerning the demographic associates are not conclusive. Older age was reported to be associated with greater use of traditional medicine in Taiwan [18, 19], but the pattern seemed not applicable in Japan [15]. Studies on the ethnic disparity are rare, limited to one study on Singapore [16], which reported that the prevalence of use of complementary and alternative medicine was higher for Chinese than for Malays and Indians.

Findings on socioeconomic factors are particularly inconsistent. A recent study [20] revealed that education could promote the understanding of traditional medicine and facilitate its use in China; in contrast, this association appeared not valid in Singapore and Japan [15, 16]. Similarly, whether income and financial status matters is unclear. Higher income was associated with more use of acupuncture use in stroke patients in Taiwan [21]; however, such association was not significant in Japan [15]; in China, lack of health insurance was correlated with higher use of traditional herbal treatments, which tend to be cheaper than conventional medicine [22].

Unlike these demographic and socioeconomic factors, relational and contextual factors of family, social network and community are often missing in literature, despite it being well established that the utilization of medical care depends on physical and social contextual factors, such as availability of infrastructure and health services of the local communities, institutional factors, social network and family support [23, 24]. Empirical evidence strongly supports that accessibility to and quality of medical services and the development level of a community are all significantly associated with individuals’ health-seeking behaviors [25, 26]. A recent report [21], for example, revealed that living in regions with a higher density of traditional medicine practitioners was positively correlated to the use of acupuncture for stroke. These important findings warrant further inquiry and development.

This paper aims to contribute to the field by systematically examining factors affecting the belief in and use of traditional Chinese medicine among older adults of Shanghai, China. As China’s major economic centre, Shanghai has long been at the nation’s forefront of healthcare system development. In recent years, the GDP per capita of Shanghai is quickly approaching 20,000 US dollars. It is also the most populous city (14.6 million household registered population in 2017) and has the highest proportion of older adults in China (33.1% age 60+ in 2017) [27]. Therefore, Shanghai represents an impending point in the trajectory of other major cities in China, and the study results can thus be potentially applied for future projections of those cities.

## Methods

### Data

The data came from Survey of Life and Opinion of Shanghai Older Adults, which was carried out in 2013 by the Shanghai Research Centre of Aging (SRCA) [28] (see the survey questionnaire of the Additional file 1). At the time of interview, informed consent was obtained from all participants. The survey employed a multistage cluster sampling procedure with a proposed sample size of 3500. First, ten districts were randomly chosen from the entire Shanghai municipality, five districts from the central urban areas (Huangpu, Xuhui, Yangpu, Zhabei, and Changning) and the other five from the suburban and rural areas (Pudong, Jiading, Minhang, Qingpu and Jinshan). Next, four street residential committees were sampled in each urban district, and three street residential committees or village committees were sampled in each suburban and rural district. Lastly, in each committee, 80 individuals aged 60 or older were randomly chosen from a complete roster of all local 60+ elders, based on the official age-sex-specific composition at the end of 2012. Another 20 individuals aged 50 to 59 were randomly sampled from the nearby residence. The final sample size is 3418.

### Measurement

We measured the belief in traditional medicine by asking “In your opinion, is the Chinese traditional medicine effective?” with answers “Yes”, “No” or “Not sure”. As the proportion of “No” is low about 5% in the survey, we combine it with “Not sure”. Regarding the use of traditional medicine, we asked “Did you ever use the Chinese traditional medicine?” with answers “Yes” or “No”.

For the basic demographic variables, we included gender, age, and location of residence (urban vs. rural). Regarding the medical factors, 12 binary variables of physician-diagnosed diseases and conditions (yes vs. no) were included: 1) hypertension, 2) cardiovascular diseases, 3) stroke, 4) pneumonia/bronchitis/pneumonectasia/asthma, 5) diabetes, 6) depression, 7) cancer, 8) prostatitis, 9) osteoporosis/osteoarthritis, 10) fracture, 11) Parkinson’s disease, and 12) nervous system disease. We used the three socioeconomic variables in analyses, education, income, and occupation. Educational attainment was classified into four categories of primary school or below, middle school, high/polytechnic school, and college or above. For income, we calculated the amount of respondents’ monthly income for the past one year. We set a cap of 10,000 to exclude nine outliers, and then used the income quantiles as its values. We asked respondents about their current (or, if they have retired, previous) primary occupation (professional/administrative, clerk/service, physical labor, and others).

We additionally examined a set of factors related to family, social network and community, including marital status, number of confidents, number of community facilities, and district-level indicators of economic development and medical support. The survey asked about whether the respondent has spouse or not, which is the most important source of family support in later life. In regards to social networks, we examined respondents on “How many confidents do you have in daily life?”, which indicates the extent of strong ties of the older adults; we also asked if respondents personally knew of any medical staff such as nurses, pharmacists, doctors in their daily life, in order to capture the influence from the general social contacts regarding the traditional medicine. For community-level factors, in the survey, we asked whether the community the respondent resided in has 1) an activity centre, 2) a cultural centre, 3) a library, 4) a gym or sport ground, 5) a senior centre, 6) a senior school, and 7) a day care centre. The total number of these community facilities was used to indicate the extent of the community physical environmental support. In addition, we included two district-level variables the GDP per capita and the number of medical staff per 10,000 residents.

### Analytical strategy

The hierarchical data of individuals clustered within districts made us to apply a multilevel logistic model for this study:

Level 1: $$ \mathit{\ln}\left(\frac{p_{ij}}{1-{p}_{ij}}\right)={\beta}_{0j}+{\beta}_1{X}_{1\mathrm{ij}}+\dots +{\beta}_k{X}_{kij}+{e}_{ij} $$

Level 2: *β*_0*j*_ = *γ*_00_ + *γ*_01_*GDP*_*j*_ + *γ*_02_*Med*_*j*_ + *u*_0*j*_

where *p*_*ij*_ is the probability of an older adult *i* at district *j* to use traditional medicine (or to believe traditional medicine); *β*_1_ to *β*_*k*_ are the coefficients for various *k* covariates; *e*_*ij*_ indicates the individual variation; *u*_0*j*_ is the variation between districts; *GDP*_*j*_ and *Med*_*j*_ separately indicate the district-level (local community) factors in terms of the GDP per capita and the number of medical professional staff per 10,000 residents; *β*_0*j*_ is the individual-level intercept, and *γ*_00_ is the district-level intercept. In modelling, we firstly included the demographic variables in the model (Model I), and then separately added variables of the major disease chronic conditions (Model II), individual-level socioeconomic variables (Model III), as well as the meso/macro-level social factors (Model IV). The full model (Model V) included all the variables as above.

Package R, function *glmer* was used to fit the generalized linear mixed models [29]. The estimation method, the adaptive Gauss-Hermite approximation to the likelihood [30], is a superior method for estimating variance components when the number of clusters is fewer than 10 clusters [31]. The number of districts for the current study is 10. The intra-class correlation coefficient (ICC) of the null model is 0.04 (use of traditional medicine) and 0.06 (belief in traditional medicine), justifying the appropriateness of the use of the multi-level models in this analysis. No imputation was conducted as all variables in analysis have a missing rate lower than 2%.

## Results

The sample characteristics used in this study are described in Table 1. About 50% of older adults in our sample believed and used traditional medicine. The entire sample had a mean age of 68.9 years, 47.6% were male, and 87.3% were urban residents. The most prevalent diseases and conditions diagnosed by doctors for these older adults were hypertension (48.0%), cardiovascular diseases (23.6%), and osteoporosis/osteoarthritis (20.7%). About 60% of them received education no more than middle school, and about half of them worked in a physical labor-related sector. Their average monthly income was 2484.2 *yuan* in 2013. Most had spouses and they had an average of about 4 persons to confide in in their daily life. About 17% of them knew one medical professional staff in their daily life. On average, their communities had about 5.3 community facilities of activity. For the 10 sampled districts, the GDP per capita was 106,000 *yuan*, and the average number of medical professional staffs per 10,000 residents in the sampled districts was around 100.

**Table 1:** Description of sample characteristics

Sample size	3418
***Belief on and Use of Traditional Medicine***	
Belief on Traditional Medicine (yes = 1, no = 0)	46.6%
Use of Traditional Medicine (yes = 1, no = 0)	45.9%
***Demographics***	
Gender (male = 1, female = 0)	47.6%
Mean age (range: 50–100)	68.9 (sd = 10.3)
Residence (urban = 1, rural = 0)	87.3%
***Medical factors***	
Hypertension (yes = 1, no = 0)	48.0%
Cardiovascular diseases (yes = 1, no = 0)	23.6%
Stroke (yes = 1, no = 0)	5.8%
Pneumonia/ … / asthma (yes = 1, no = 0)	11.9%
Diabetes (yes = 1, no = 0)	11.1%
Depression (yes = 1, no = 0)	1.1%
Cancer (yes = 1, no = 0)	2.5%
Prostatitis (yes = 1, no = 0)	5.0%
Osteoporosis/osteoarthritis (yes = 1, no = 0)	20.7%
Fracture (yes = 1, no = 0)	4.6%
Parkinson’s disease (yes = 1, no = 0)	0.7%
Nervous system disease (yes = 1, no = 0)	5.5%
***Socioeconomics***	
Education: Primary school or below	28.0%
Middle school	32.8%
High/polytechnic school	25.7%
Colleague or above.	13.4%
Monthly income (range: 0–10,000 Yuan)	2484.2
Main occupation: Professional/administrative	21.0%
Clerk/service	19.5%
Physical labor	46.8%
Other	12.8%
***Relational and contextual factors***	
Spouse accompany (yes = 1, no = 0)	77.8%
Average number of strong ties to confide	4.0 (sd = 3.8)
Knowing medical staff (yes = 1, no = 0)	17.0%
Average number of community facilities	5.3 (sd = 2.3)
GDP per capita in the district (unit: 10,000 Yuan)	10.6 (sd = 4.5)
Medical staff per 10,000 residents in the district	97.6 (sd = 66.2)

To map out the sociodemographic profile of these believers and users, we further examined all these sample characteristics by belief in and use of traditional medicine. As can be seen from Table 2, believers and users of traditional medicine in Shanghai were more likely to be female, married, and urban residents. According to these unadjusted statistics, believers and users of traditional medicine tended to have a lower education and have a primary job category when they were in prime labor force ages related to physical laboring or clerk/service. In regard to their medical conditions, about a half of believers and users had hypertension, about 25% of them had cardiovascular disease, and about 20% of them had osteoporosis/osteoarthritis.

**Table 2:** Sample characteristics by attitude and use of traditional medicine

	Belief on Traditional Medicine		Use of Traditional Medicine	
	Yes	No	Yes	No
**Sample size**	1593	1825	1568	1850
***Demographics***				
Gender (male = 1, female = 0)	46.3%	48.7%	43.8%	50.8%
Mean age (range: 50–100)	68.5	69.3	69.5	68.5
Residence (urban = 1, rural = 0)	85.2%	89.2%	85.4%	88.9%
***Medical factors***				
Hypertension (yes = 1, no = 0)	47.0%	49.0%	50.5%	46.0%
Cardiovascular diseases (yes = 1, no = 0)	24.0%	23.2%	27.9%	19.9%
Stroke (yes = 1, no = 0)	5.2%	6.4%	6.1%	5.5%
Pneumonia/ … / asthma (yes = 1, no = 0)	11.9%	12.0%	15.2%	9.1%
Diabetes (yes = 1, no = 0)	10.7%	11.5%	12.1%	10.3%
Depression (yes = 1, no = 0)	1.1%	1.2%	1.3%	1.0%
Cancer (yes = 1, no = 0)	3.5%	1.6%	4.0%	1.2%
Prostatitis (yes = 1, no = 0)	6.2%	4.0%	6.3%	3.9%
Osteoporosis/osteoarthritis (yes = 1, no = 0)	20.0%	21.3%	24.0%	17.9%
Fracture (yes = 1, no = 0)	5.5%	3.9%	6.1%	3.4%
Parkinson’s disease (yes = 1, no = 0)	0.4%	0.9%	0.6%	0.8%
Nervous system disease (yes = 1, no = 0)	4.8%	6.1%	7.3%	4.1%
***Socioeconomics***				
Education: Primary school or below	25.3%	30.4%	27.7%	28.3%
Middle school	30.2%	35.1%	30.5%	34.8%
High/polytechnic school	27.9%	23.8%	25.8%	25.7%
Colleague or above.	16.6%	10.7%	16.1%	11.2%
Monthly income (range: 0–10,000 Yuan)	2.6	2.4	2.5	2.5
Main occupation: Professional/administrative	25.4%	17.1%	24.2%	18.2%
Clerk/service	19.9%	19.1%	17.9%	20.8%
Physical labor	42.8%	50.3%	44.5%	48.7%
Other	12.0%	13.6%	13.4%	12.4%
***Relational and contextual factors***				
Spouse accompany (yes = 1, no = 0)	80.2%	75.7%	78.1%	77.5%
Average number of strong ties to confide	4.3	3.7	4.2	3.7
Knowing medical staff (yes = 1, no = 0)	21.0%	13.5%	19.1%	15.2%
Average number of community facilities	5.4	5.2	5.4	5.2
GDP per capita in the district (unit: 10,000 Yuan)	1.4	1.5	1.5	1.4
Medical staff per 10,000 residents in the district	10.8	10.7	10.9	10.6

Table 3 summarizes the odds ratios of correlates of using traditional medicine (for crude odds ratios of all variables and the 95% confidence intervals, see Table 3A to 3F in supplementary materials of the Additional file 2). As can be seen, males were less likely to use traditional medicine than females. Older ages seemed to positively relate to more use of traditional medicine, but the association disappeared when the major diseases and conditions are controlled for. Rural residents were more likely to use traditional medicine than their urban counterparts. It is interesting to note that cardiovascular diseases, lung diseases, cancer, prostatitis, arthritis, and nervous system diseases were positive correlates for using traditional medicine. Better education and white-collar occupation promoted the use of traditional medicine. Among relational and contextual factors, number of strong ties to confide was the only significant factor and was positively associated with the use of traditional medicine.

**Table 3:** Odds ratios of correlatives for use of traditional medicine based on multi-level logit regression models

	Model I	Model II	Model III	Model IV	Model V
***Demographics***					
Gender (male = 1, female = 0)	0.762***	0.752***	0.690***	0.754***	0.681***
Age (range: 50–100)	1.007*	0.998	1.010*	1.012**	1.005
Residence (urban = 1, rural = 0)	0.712*	0.715*	0.619**	0.763	0.682*
***Medical factors***					
Hypertension (yes = 1, no = 0)		1.166*			1.138
Cardiovascular diseases (yes = 1, no = 0)		1.447***			1.446***
Stroke (yes = 1, no = 0)		0.947			0.991
Pneumonia/ … / asthma (yes = 1, no = 0)		1.525***			1.559***
Diabetes (yes = 1, no = 0)		1.156			1.188
Depression (yes = 1, no = 0)		1.454			1.540
Cancer (yes = 1, no = 0)		3.643***			3.391***
Prostatitis (yes = 1, no = 0)		1.712**			1.605**
Osteoporosis/osteoarthritis (yes = 1, no = 0)		1.300***			1.318**
Fracture (yes = 1, no = 0)		1.452*			1.398
Parkinson’s disease (yes = 1, no = 0)		0.560			0.572
Nervous system disease (yes = 1, no = 0)		1.389			1.446*
***Socioeconomics***					
Education (primary school or below = 0)					
Middle school			1.175		1.206
High/polytechnic school			1.341*		1.382*
Colleague or above.			1.724***		1.753***
Monthly income (range: 0–10,000 yuan)			1.022		1.017
Main occupation (professional/administrative = 0)					
Clerk/service			0.740*		0.778*
Physical labor			0.853		0.902
Other			0.829		0.910
***Relational and contextual factors***					
Spouse accompany (yes = 1, no = 0)				1.141	1.129
Number of strong ties to confide				1.049***	1.044***
Knowing medical staff (yes = 1, no = 0)				1.191	1.085
Number of community facilities				1.026	1.022
GDP per capita in the district (unit: 10,000 yuan)				1.000	1.049
Medical staff per 10,000 residents in the district				1.022	1.008
Individual-level Intercept	0.785	1.001	0.712	0.268**	0.330*
Log Likelihood	− 2307.47	− 2250.28	− 2285.15	− 2289.81	− 2217.10
Variance of District-level Intercept	0.12	0.12	0.11	0.13	0.12
Sample size	3415	3415	3408	3415	3408

*** *p* < 0.001, ** *p* < 0.01, * *p* < 0.05

The results on the belief over traditional medicine showed similar associational patterns, as seen in Table 4 (for crude odds ratios of all variables and the 95% confidence intervals, see Table 4A to 4F in supplementary materials of the Additional file 2). However, there are some notable differences. Only older adults who had a cancer, a prostatitis, or a fracture tended to believe in the effect of traditional treatment. And both the number of strong ties to confide and knowing medical staff significantly promoted belief in the traditional medicine.

**Table 4:** Odds ratios of correlatives for belief on traditional medicine based on multi-level logit regression models

	Model I	Model II	Model III	Model IV	Model V
***Demographics***					
Gender (male = 1, female = 0)	0.895	0.839*	0.790**	0.888	0.758***
Age (range: 50–100)	0.991**	0.990**	0.995	0.997	0.999
Residence (urban = 1, rural = 0)	0.737*	0.687**	0.530***	0.805	0.579***
***Medical factors***					
Hypertension (yes = 1, no = 0)		0.984			0.950
Cardiovascular diseases (yes = 1, no = 0)		1.160			1.143
Stroke (yes = 1, no = 0)		0.838			0.886
Pneumonia/ … / asthma (yes = 1, no = 0)		1.020			1.042
Diabetes (yes = 1, no = 0)		0.968			1.006
Depression (yes = 1, no = 0)		1.253			1.373
Cancer (yes = 1, no = 0)		2.100**			1.823*
Prostatitis (yes = 1, no = 0)		1.740**			1.628**
Osteoporosis/osteoarthritis (yes = 1, no = 0)		0.846			0.848
Fracture (yes = 1, no = 0)		1.511*			1.440*
Parkinson’s disease (yes = 1, no = 0)		0.550			0.556
Nervous system disease (yes = 1, no = 0)		0.706*			0.743
***Socioeconomics***					
Education (primary school or below = 0)					
Middle school			1.210		1.225
High/polytechnic school			1.499**		1.485**
Colleague or above.			1.702***		1.618**
Monthly income (range: 0–10,000 yuan)			1.066		1.053
Main occupation (professional/administrative = 0)					
Clerk/service			0.774		0.820
Physical labor			0.729		0.767*
Other			0.649		0.733
***Relational and contextual factors***					
Spouse accompany (yes = 1, no = 0)				1.161	1.078
Number of strong ties to confide				1.054***	1.052***
Knowing medical staff (yes = 1, no = 0)				1.540***	1.420***
Number of community facilities				1.023	1.015
GDP per capita in the district (unit: 10,000 yuan)				0.981	1.031
Medical staff per 10,000 residents in the district				1.018	1.002
Individual-level Intercept	2.181*	2.567**	2.018	0.695	0.911
Log Likelihood	− 2288.98	− 2269.16	− 2252.12	− 2259.44	− 2215.69
District-level Intercept	0.21	0.20	0.18	0.25	0.22
Sample size	3415	3415	3408	3415	3408

*** *p* < 0.001, ** *p* < 0.01, * *p* < 0.05

## Discussion

The World Health Organization recently called for the integration of traditional medicine into countries’ national health system [32]. In this regard, it is imperative to understand varied perceptions on and use styles of traditional medicine. In practice, it is long noted that patients tend to avoid consulting or informing physicians about their use of traditional medicine [15, 33]. Such omitted information may lead to negative outcomes in diagnosis and treatment, a public health concern that may be mitigated by information on the demographic and socioeconomic factors that influence traditional medicine use. Moreover, for societies where traditional medicine still plays a crucial role in health system, a better understanding of practice of traditional medicine will also facilitate the development of an equitable and efficient public health system [18, 34].

This study contributed to the literature by focusing on the practice of traditional medicine amongst older adults. We found the use of and the belief in traditional medicine were prevalent amongst Shanghai older adults. Although some scholars recently reported a decline in utilization of traditional medicine in China [10, 35], the trend might be reversed soon, especially amongst individuals of old ages. Unprecedented worldwide population aging has caused rising prevalence of chronic diseases such as cardiovascular disease, cancer, diabetes, and chronic respiratory disease, which are on track to become the major causes of disability and mortality. In this setting, traditional medicine presents an opportunity in that it is praised for having an advantage in chronic disease treatment and prevention over conventional medicine [36, 37]. A rising market for traditional medicine driven by population aging has long been foreseen [38, 39], and many Asian nations have already recognized and responded to this. For example, a recent policy initiative by the State Council of China stressed that developing traditional medicine would play an essential role in meeting the demands of eldercare services. Similarly, responding to the broad adoption of traditional medicine by older patients, the Japan Geriatrics Society recently released official guidelines for the use of traditional medicine [9].

In response to the trend to study practice of traditional medicine through the KAP model, both belief in and use of traditional medicine were examined in this study. As observed, there were no major differences in term of their associational patterns with various covariates, suggesting good consistency in the cognitive and behavioral aspects of utilizing traditional medicine amongst Shanghai seniors. However, our further analyses revealed that there were about 11% of our respondents who had faith in traditional medicine but never used it and about 12% of respondents who used traditional medicine but held little belief. To understand these interesting types of respondents are a good research direction for future studies.

This study also added to the literature by investigating the associations of multiple diseases with traditional medicine for the general population of old age. This is different from numerous studies that focused on patients of specific diseases, such as cancer [40, 41], stroke [21, 42], and arthritis [34], or on specific treatment such as acupuncture [43]. The disease-specific studies are clinically oriented, which examine factors affecting the use of traditional medicine among certain patients in order to inform and facilitate physicians for diagnosis and treatment in conventional medicine. However, this research is based on the general population, which could help reveal to what extent having a specific medical issue could trigger the use of traditional medicine among older adults [44, 45].

Along this line, we found some significant associations of chronic diseases and conditions with belief in and the use of traditional medicine, which may capture important lay perceptions and cultural practices towards traditional medicine in China. The academic and professional tensions between traditional and conventional medicine are hard to settle [46]. However, in Asia, the belief and use of traditional medicine have deep cultural and spiritual bases [47, 48], with or without scientific backups. In this study, we found out that people were more likely to utilize the traditional medicine when they had cardiovascular diseases, lung diseases, cancer, prostatitis, arthritis and nervous system diseases; at the same time, people with cancer, prostatitis and fracture were highly likely to believe in the effect of traditional treatment.

Multiple morbidities are common among older adults, it is thus meaningful to examine how combinations of disease and condition, instead of single disease or condition, are associated with use and belief of traditional medicine among seniors. To explore this issue, we added a new variable of the number of disease and condition in the regression analyses (see Table A in supplementary materials of the Additional file 2), and found that the number of disease/condition was positively related to use and belief of traditional medicine among seniors, though such association was only significant for use of medicine. To further address the issue of multiple morbidities, we developed a list of major types of disease and condition based on their prevalence, and then ran regression analyses with the variable of types of disease and condition (see Table B in supplementary materials of the Additional file 2). These results interestingly suggested that use of traditional medicine of seniors with hypertension mostly occurred when the hypertension came with cardiovascular diseases, osteoporosis and/or diabetes.

We had intriguing findings about socioeconomic factors, for which a duality seemed to exist. On the one hand, we found that educational achievement to be positively related to the utilization of traditional medicine. Though results on income and occupational status were not consistently significant, our further analyses based on the overall socioeconomic status (SES) index revealed that seniors of low SES were significantly less likely to use traditional medicine than those of high SES. The pattern is in line with previous findings that the traditional medicine were used by those with more resources and better education [20, 21], which seems to suggest that traditional medicine is likely adopted as complementary to the conventional medicine by those with socioeconomic advantages in China. At the same time, however, we observed that the rural older people were more likely to use and believe in traditional medicine than their urban counter parts, despite being socioeconomically disadvantaged. Such a major rural-urban disparity is also acknowledged by some Western literature, commonly explained by the fact that rural areas have lower access to conventional medicine [49, 50]. Traditional medicine has long served as a crucial alternative in Asian for those who cannot access formal care [22]. Therefore, besides the scenario that traditional medicine is used as complementary medicine for socioeconomically advantaged individuals, it could also be also possible that it serves as alternative medicine for socioeconomically disadvantaged. Due to a major limitation of this study that we did not examine the use of conventional medicine of respondents, the findings on such socioeconomic patterns should not be conclusive, but more of a meaningful hypothesis to be tested by future studies. Interestingly, in support of these patterns, a recent study in Chongqing, China [45], reported that along the four levels of education and income, both the highest and lowest socioeconomic groups showed strong preference for traditional medicine.

Concerning macro-level factors such as GDP per capital, we did not find significant associations. This may be due to the relative homogeneity of the healthcare resources throughout Shanghai, and/or may be because Shanghai residents are sufficiently mobile to seek healthcare beyond and are thus unrestrained by the resources of their own immediate district. However, we observed significant positive impacts from social network, which is in accordance with findings from the West [51, 52]. Our results implied that social ties may channel medical information and provide financial support to facilitate the use of traditional medicine. In particular, connections with medical professionals may effectively increase trust of the old adults over the traditional medicine. Medical experts usually play an essential role in affecting health seek behaviours, especially among those of low social economic status, for which our study provides supporting evidence in regard to the transitional medicine.

As exploratory research on this topic, this study had some limitations. When we asked respondents about traditional medicine, we did not distinguish any types of traditional Chinese medicine among herb treatment, nutritional intake, acupuncture, or Tai-chi. For the users of traditional Chinese medicine, we also did not know the timing and the duration of their use, as well as the background of using (for example, whether it was prescribed in clinic or directly purchased from pharmacy). In addition, we also did not ask about the use of conventional medicine, the information of which will surely help understand whether the traditional medicine is used in a complementary or alternative manner. More efforts are certainly needed in the future data collection. Moreover, although we examined a series of correlates of traditional medicine, the study was not able to reveal their causality with the belief in and use of traditional medicine. This is especially the case for the factors of the diseases and conditions. As we did not ask the type of disease the traditional medicine was used for, it was hard to tell if the specific medical issues or concerns exactly led to the choices of using traditional medicine. Future studies, particularly those of longitudinal design, are thus warranted to examine this issue.

## Conclusion

Using a cross-sectional survey, this study revealed that the prevalence rates of belief and use of traditional medicine were both close to 50% among amongst Shanghai older adults. These high rates suggest a great potential of traditional medicine to address the increasing healthcare needs due to population aging. We also found patients of cardiovascular diseases, lung diseases, cancer, prostatitis, arthritis and nervous system diseases were more likely to seek help from traditional medicine. Though not conclusive, our study suggested that traditional medicine in China appears to serve two distinct functions: complementary medicine for socioeconomically advantaged individuals, and alternative medicine for socioeconomically disadvantaged.

## Supplementary information

**Additional file 1.** Survey questionnaire.

**Additional file 2.** Supplementary materials.

## Data Availability

The datasets used and/or analyzed during the current study are available from the corresponding author on reasonable request.

## Abbreviations

GDP: Gross Domestic Product
ICC: The intra-class correlation coefficient
KAP: The Knowledge, Attitude and Practice model
SES: The socioeconomic status
SRCA: Shanghai Research Centre of Aging
