# Supplementary material for: Belief in and use of traditional Chinese medicine in Shanghai older adults: a cross-sectional study
Source: BMC Complement Med Ther. 2020 Apr 28;20:128. doi: 10.1186/s12906-020-02910-x (PMC7189641; doi:10.1186/s12906-020-02910-x)
Supplement: Supplementary file 1 — Additional file 1. Survey questionnaire. [file 12906_2020_2910_MOESM1_ESM.pdf]

The Survey of Life and Opinion on Shanghai Older Adults

Survey Number：_____________

________District（County）_________Sub-district（Town）________ Residential (Village) Committee
________Road（Street, Lane ________Building (No.) ________Room

Name：____________________ Contact Number: __________________

*********************** Preface **********************

Dear Sir/Madam,

Hi. I am an interviewer of the Shanghai Research Centre of Aging (SRCA). Since 1998, we have been collecting the demographic and social information and opinions of the elderly in urban and rural areas of Shanghai, in order to improve the living standard of Shanghai seniors and contribute to the research on aging. As this investigation is significant to the aging research and the eldercare policymaking in Shanghai, we look forward to the cooperation of you and your family. We are trying to know your true opinions. We solemnly promise that all information collected from this interview would remain strictly confidential, and the data will be only used for scientific research. Your private information such as name, address and phone number will certainly not be included in the data. Thank you very much for your help and support!

Wish you healthy and happy,
Shanghai Research Centre of Aging

Interview Record

Date of Interview		Start Time	End Time	Reason for not completing the survey			
Month	Day			Refuse	Not at home	Migration	Others

Signature of Investigator (Stamp)：__________________ Date:______Year______Month_______Day

Signature of Instructor (Stamp)：___________________ Date______ Year_______Month_______Day

Signature of Inspector (Stamp)___________________ Date______ Year_______Month_______Day

In general, the questionnaire must be answered by the respondent himself/herself. It may be answered by someone else under certain circumstances. In this case, the answers should be marked by the investigator as "answered by someone else" next to the questions. The order of people could answer for the respondent is family numbers, neighbors or friends. If no one can help to answer, please note the reasons beside the questions.

**A. Basic Information**		**Code**
**A1. Gender:**	1. Male 2. Female	□
**A2. Current Marital Status:**	1.Never Married<br>2. First Marriage and living with spouse **(Please skip to A3)**<br>3. Remarriage and living with spouse **(Please Skip to A3)**<br>4. Non-marriage cohabitation **(Please skip to A3)**<br>5. Divorced 6. Separated 7. Widowed	□
A2a.Do you want a partner?	1. Yes 2. No 3. I have no idea 4.Refuse to answer	□
**A3. Your Birthday:**<br>**A3a.Your spouse's birthday:**	______ Year ____ Month<br>_______Year_____Month	19□□/□□<br>19□□/□□
**A4. Type of your household:**	1. Non-agricultural 2. Agricultural	□
**A5. Your political status:**	1.Party member of the CPC<br>2. Member of democratic parties<br>3. Citizen	□
**A6. Your educational degree:**<br>**A6a.Your spouse's educational degree:**	1.Below primary school 2. Primary school<br>3. Middle school 4. High school<br>5. Technical secondary school/Polytechnics/ Vocational school<br>6. Diploma 7.Bachelor<br>8. Master 9. PhD<br>10.Others（Please specify） ____________	□□<br>□□
**A7. Your religious belief:**<br>**A7a.Your spouse's religious belief:**	1.No religious belief (**Please skip to A8)** 2. Buddhism<br>3. Taoism 4.Islam 5.Catholicism<br>6.Non-catholic Christian 7. Others（Please specify） ______	□<br>□

A7b. Do you regularly attend religious activities?	1.Never 2. Once a few months<br>3.About once a month 4. About once a week<br>5. About once a day 6.Others（Please specify）______	□
**A8. How many children have you raised in total?**	1.Son____ 2.Daughter____	□<br>□
**A9. Who are you living with?**	1. Alone 2. Only with spouse<br>3. Only with a child (or children)<br>4. With spouse and a child (or children)<br>5. Nursing home/apartment for the elderly<br>6. Others（Please specify）______	□
A9a.Who would you like to live with?	1. Alone 2. Only with spouse<br>3. Only with a child (or children)<br>4. With spouse and a child (or children)<br>5. Nursing home/apartment for the elderly<br>6. Others（Please specify）______	□
**A10.Have your children provided home care for you in the past year?**	1.Yes（_________days） 2.No<br>3. I do not need home care	□/□□□
**A11.Have your children given you money in the past year?**	1.Yes（_________yuan） 2.No<br>3. I do not need money from my children	□/□□□□□
**A12.What kind of building do you live in?**	1. Multi-storied residential housing 2. High-rise housing<br>3. Garden House 4. Shikumen housing<br>5.Two-story or three-story house in countryside<br>6. Bungalows 7. Makeshift House<br>8.Others（Please specify）_______	□
A12a.When was your house built?	Year ____________	□□<br>□□
A12b. The property of your house belongs to	1. You and (or) your spouse<br>2. Your children and (or) their spouse<br>3. Your grandchildren and (or) their spouse<br>4. Rental<br>5.Others (please specify) __________	□
A12c.How big is your house?	The total living space is _________㎡<br>The living space per capita is _____㎡	□□□<br>□□□

B. Financial Status		Code
**B1. Have you retired?**<br>**B1a.Have your spouse retired?**	1. Retired 2. Retired with honour<br>3. Not yet 4. Not applicable **(Please skip to B4)**	☐<br>☐
**B2. What was (is) you occupation?**<br>**B2a. What was (is) your spouse's occupation?**<br>**（Please fill in the previous occupation if you or your spouse is retired; please fill in the current occupation if not yet.）**	1.Professional/Doctor/Teacher<br>2.Administator<br>3.Staff and related personnel<br>4.Employee in commercial and service industry<br>5.Agriculture, forestry, animal husbandry and fishery<br>6.Worker in production, transport or related personnel<br>7.Other worker who is not able to classify (specify____)<br>8.Unemployed	☐<br>☐
**B3. What is the nature of your previous(current) work unit？**<br>**B3a.What is the nature of your spouse's previous (current) work unit?**<br>**（Please fill in the nature of previous work unit if you or your spouse is retired, please fill in the nature of current work unit if not yet.）**	1.Party and government organization<br>2. Public Institutes 3. State-owned enterprises<br>4. Private enterprises 5. Foreign-funded enterprises<br>6.Rural autonomous organizations<br>7.Others（Please specify）_________	☐<br>☐
**B4. Are you currently engaged in a gainful employment?**<br>**B4a. Is your spouse currently engaged in a gainful employment？**	1.Yes<br>2. No but look forward to<br>3. No and do not want to **(Please skip to B6)**	☐<br>☐
**B5. What is the main purpose of the gainful employment you are currently engaged in or hoping to be engaged in?**<br>**（According to the degree of importance, you can choose up to three items, fill in 1, 2, 3 to the brackets before items. If you cannot answer ,please choose No.9.）**	( ) 1.To supplement your own living expenses<br>( ) 2.To subsidize children's /grandchildren's expense<br>( ) 3. To make life more meaningful<br>( ) 4. Good for your health<br>( ) 5. Others (Please specify) _________<br>( ) 9. Cannot answer.	First:<br>☐<br>Second:<br>☐<br>Third:<br>☐<br>☐

**B6. What kind of pension security do you have?** **(Multiple choice, please tick the corresponding options. If you do not have any insurance, please tick No.11.Please fill in 1 to all items you chosen, and fill in 0 to others.)**	( )1. Basic endowment insurance for urban workers<br>( )2. Basic endowment insurance for urban residents<br>( )3. Small town basic endowment insurance<br>( )4. Rural social endowment insurance<br>( )5.Complementary corporate pension insurance<br>( )6.Commercial endowment insurance<br>( )7. Land acquisition pension<br>( )8. Rural pension subsidy<br>( )9. Old-age insurance<br>( )10. Social assistance<br>( )11. Others (please specify) _______<br>( )12. I do not have any old-age security	

**B7. How much was your personal income per month in average in last year** （**If there was some object in your income, you need to convert it into cash.** ）	1. Pension	yuan	
	2. Pension subsidy	yuan	
	3. Income from work not retried yet	yuan	
	4. Income from work after retired	yuan	
	5. Rental	yuan	
	6. Financial investment income	yuan	
	7. Subsidies from children and grandchildren	yuan	
	8. Subsidies from other relatives	yuan	
	9. Social assistance	yuan	
	10. Other income (please specify)	yuan	

B7a.What are the main sources of income	**The main sources of income are_______ (Please fill in with numbers according to B7)**	☐
**B8. What is the current financial situation of your family**	1.Very well-off<br>2.Well-off<br>3.Average<br>4.Poor<br>5.Very poor	☐
B8a. Compared to five years ago, has the financial situation of your family changed?	1. Improved a lot<br>2. Improved slightly<br>3. Similar<br>4. Decline slightly<br>5. Declined a lot	☐

C. Health Status		Code
**C1. Has your doctor ever diagnosed you with any following health problems?**<br><br>**(multiple choice, please tick at corresponding options)**	( )1. Hypertension<br>( )2. Cardio cerebrovascular diseases such as CHD<br>( )3. Cerebral stroke<br>( )4. Bronchitis，emphysema，asthma，pneumonia<br>( )5. Diabetes ( )6. Depression<br>( )7. Cancer ( )8. Prostatitis<br>( )9. Bone or joint problems such as osteoporosis and arthritis<br>( )10. Fracture ( )11. Parkinson's disease<br>( )12. Nervous system disease<br>( )13.Others（Please specify）___________	
**C2. How did you feel about your health in the past week**	1.Very good<br>2. Good<br>3. Normal<br>4. Bad<br>5.I have no idea.	☐
**C3. How have you felt in the past week?**		
a. Are you satisfied with the present life	1. Yes 2. No 3. I have no idea	☐
b. Do you feel empty in your life	1. Yes 2. No 3. I have no idea	☐
c. Do you always feel happy	1. Yes 2. No 3. I have no idea	☐
d. Do you always feel lonely	1. Yes 2. No 3. I have no idea	☐
e. Do you think most people live better than you?	1. Yes 2. No 3. I have no idea	☐
**C4. How often is it that you …during the last 3 months.**		
a. exhausted without increasing activity.	1.Never 2. occasionally 3. often 4. Very often 5. always	☐
b. had fatigue could be alleviated by rest.	1.Never 2. occasionally 3. often 4. Very often 5. always	☐
c. were lethargic when working	1.Never 2. occasionally 3. often 4. Very often 5. always	☐
d. suffered from headaches	1.Never 2. occasionally 3. often 4. Very often 5. always	☐
e. suffered from dizziness	1.Never 2. occasionally 3. often 4. Very often 5. always	☐
f. eyes ached	1.Never 2. occasionally 3. often 4. Very often 5. always	☐
g. suffered from a sore throat	1.Never 2. occasionally 3. often 4. Very often 5. always	☐
h. muscles or joints felt stiff	1.Never 2. occasionally 3. often 4. Very often 5. always	☐
i. had pain in your shoulder/neck/waist	1.Never 2. occasionally 3. often 4. Very often 5. always	☐

j. had heavy feeling in legs when walking	1.Never 2. occasionally 3. often 4. Very often 5. always	☐
k. felt out of breath while sitting still	1.Never 2. occasionally 3. often 4. Very often 5. always	☐
l. suffered from chest congestion	1.Never 2. occasionally 3. often 4. Very often 5. always	☐
m. were bothered by heart palpitations	1.Never 2. occasionally 3. often 4. Very often 5. always	☐
n. appetite was poor	1.Never 2. occasionally 3. often 4. Very often 5. always	☐
o. suffered from heartburn	1.Never 2. occasionally 3. often 4. Very often 5. always	☐
p. suffered from nausea	1.Never 2. occasionally 3. often 4. Very often 5. always	☐
q. could not tolerate the cold	1.Never 2. occasionally 3. often 4. Very often 5. always	☐
r. had difficulty falling asleep	1.Never 2. occasionally 3. often 4. Very often 5. always	☐
s. had trouble with waking up during night	1.Never 2. occasionally 3. often 4. Very often 5. always	☐
t. had trouble with your short-term memory	1.Never 2. occasionally 3. often 4. Very often 5. always	☐
u. could not respond quickly	1.Never 2. occasionally 3. often 4. Very often 5. always	☐
v. had difficulty concentrating	1.Never 2. occasionally 3. often 4. Very often 5. always	☐
w. were distracted for no reason	1.Never 2. occasionally 3. often 4. Very often 5. always	☐
x. felt nervous or jittery	1.Never 2. occasionally 3. often 4. Very often 5. always	☐
**C5. Have you caught cold in the past year?**	1.Never 2. occasionally 3. often 4. Very often 5. always	☐

D. Health behavior and medical condition — Code

D1. We would like to know about your usual medical examination. Please answer the following questions for each item

a. Have you ever done the medical examination listed

1. Yes 2. No **(please skip to Part E)** 3. I have no idea

b. If yes, when was the last time?

1. This year 2. Last year 3. The year before 4. 3 years ago

c. Where did you do the medical examination

1. Infirmary in Residential/Village Committee 2. Subdistrict/ township medical institution
3. District (County) medical institution 4. Municipal -level medical institution
5. Private hospital 6. Private clinic 7. Others (please specify) _______________

d. Did your medical insurance cover the medical examination

1.Yes 2. No 3. I have no idea

e. Do you think it is helpful to keep healthy?

1. Not helpful at all 2. Not helpful 3. Helpful 4. Very helpful 5. I have no idea

Item	a. Have you done?	b. When?	c. Where?	d. Covered by medical insurance?	e. Is it helpful?	
1. Blood routine	a1 ☐	b1 ☐	c1 ☐	d1 ☐	e1 ☐	
2. Urine routines	a2 ☐	b2 ☐	c2 ☐	d2 ☐	e2 ☐	
3. Stool test for colon cancer	a3 ☐	b3 ☐	c3 ☐	d3 ☐	e3 ☐	
The following two medical examinations are for female respondents to answer only						
4. Pap test	a4 ☐	b4 ☐	c4 ☐	d4 ☐	e4 ☐	
5. Mammography	a5 ☐	b5 ☐	c5 ☐	d5 ☐	e5 ☐	

**D2. What kind of medical insurance are you entitled to currently? (Select up to two items, please tick the corresponding options)**	( )1. Basic medical insurance for urban workers<br>( )2. Basic medical insurance for urban residents<br>( )3. Medical insurance in small town social insurance<br>( )4. Medical insurance for land-expropriated farmers<br>( )5. rural cooperative medical care<br>( )6. Health care for cadres<br>( )7. Commercial medical insurance<br>( )8. Self-pay<br>( )9. Others(please specify)___________	☐<br>☐
**D3. Who pays for your current medical expenses**	1. Free medical service<br>2. Social medical insurance<br>3. Commercial medical insurance<br>4. State and collective subsidies<br>5. Self or spouse<br>6. Children/grandchildren<br>7. Others (please specify)___________	☐
**D4. If you are sick, can you get treatment in time?**	1. Yes 2. No	☐
**D5. Where do you see a doctor**	1. Infirmary in Residential/Village Committee<br>2. Subdistrict/ township medical institution<br>3. District (County) medical institution<br>4. Municipal -level medical institution<br>5. Private hospital<br>6. Private clinic<br>7. Others (please specify)______________	☐

**D6. Do you smoke**	1. Yes, ___ per day 2. No	□/□ □
D6a. If yes, how many days did you smoke in the past 30 days	__________ Day(s)	□□
**D7. In the past few weeks, have you done any physical fitness or leisure activities that cause shortness of breath or rapid heartbeat for at least ten minutes （such as brisk walk）**	1. Yes, ___day(s) per week<br>2. No	□/□
**D8. What are your usual hobbies (Multiple choices, please tick the corresponding option)**	( )1. Reading ( )2. Writing and Painting<br>( )3. Playing chess and cards ( )4. Planting flowers and raising birds<br>( )5. Listening to music and playing an instrument<br>( )6. Watching TV ( )7. Watching movies<br>( )8. Singing and dancing ( )9. Performing opera<br>( )10. Physical Exercise ( )11. going to the park<br>( )12. Collection and Appreciation ( )13. Photography<br>( )14. Travelling ( )15. Fishing<br>( )16. Chatting ( )17. Others_____<br>( )0. No hobby	□

**E. Health Concept**		**Code**
**E1. Do you agree with the following opinion?**<br>1. Strongly agree 2. Slightly agree 3. Slightly disagree 4. Strongly disagree 5. I do not know		
a. It is difficult for me to find time for a medical examination	1. 2. 3. 4. 5.	□
b. If going for medical examination, it is difficult to find someone take care of my family (such as babysitting, cooking)	1. 2. 3. 4. 5.	□
c. It is difficult to get someone accompany me for a medical examination.	1. 2. 3. 4. 5.	□
d. Life and death are decreed by fate. We cannot change our destiny	1. 2. 3. 4. 5.	□
e. The critical illness is fated, and we cannot avoid it, such as cancer.	1. 2. 3. 4. 5.	□
f. If the cancer is destined, we cannot change it.	1. 2. 3. 4. 5.	□
g. If I do not die from this disease, I will die from other disease. Hence, it is not necessary to do a medical examination.	1. 2. 3. 4. 5.	□
h. If I feel well, there is no need to do a medical examination.	1. 2. 3. 4. 5.	□
i. Many diseases are avoidable, so we should try our best to avoid these diseases.	1. 2. 3. 4. 5.	□
j. My health strongly depends on how I take care of myself.	1. 2. 3. 4. 5.	□
k. There is no need to do a preventive medical examination for my age.	1. 2. 3. 4. 5.	□

l. A medical examination can help me find out the disease in early stage.	1. 2. 3. 4. 5.	☐
m. If the disease can be found in early stage, the recovery chance will be higher.	1. 2. 3. 4. 5.	☐
n. The medical examinations can help me stay healthy and energetic longer	1. 2. 3. 4. 5.	☐
o. The medical examination is meaningless, as I cannot afford medical expenses even if I find out any diseases	1. 2. 3. 4. 5.	☐
p. I do not believe the results of medical examination.	1. 2. 3. 4. 5.	☐

**E2. Have you had any traditional Chinese Medicine treatment?**	1.Yes 2.No	☐
E2a. Do you think Traditional Chinese Medicine is effective?	1. Yes 2. No 3. I have no idea	
**E3. Do you take health supplements regularly?**	1. Yes 2. No 3. I have no idea	☐
E3a. Do you think health supplements are good for your health?	1. The benefits are obvious<br>2. There are certain benefits for health.<br>3. The benefits are not obvious.<br>4. There is no benefit for health.<br>5. I have no idea.	☐

**F. Elderly Care Status and Willingness**		**Code**
**F1. How is your current self-care ability? (if fully unable of taking care of yourself, please fill in from which age)**	1. Fully capable of taking care of myself (please skipF6)<br>2. Partially capable of taking care of myself<br>3. Fully unable of taking care of myself (from __ years old)	☐<br>☐☐
**F2. Are you able to take care of yourself in the following basic daily activities?**		
a. Bathing	1. I can take care of myself without help<br>2. I can take care of myself, but need some help<br>3. I am not able to take care of myself	☐
b. Dressing	1. I can take care of myself without help<br>2. I can take care of myself, but need some help<br>3. I am not able to take care of myself	☐
c. Going to the toilet (can use walking stick or wheelchair)	1. I can take care of myself without help<br>2. I can take care of myself, but need some help<br>3. I am not able to take care of myself	☐

d. Move indoors (can use walking stick)	1. I can take care of myself without help<br>2. I can take care of myself, but need some help<br>3. I am not able to take care of myself	☐
e. Controlling bowel movements	1. I can take care of myself without help<br>2. I can take care of myself, but need some help<br>3. I am not able to take care of myself	☐
f. Feeding	1. I can take care of myself without help<br>2. I can take care of myself, but need some help<br>3. I am not able to take care of myself	☐
**F3. If you need help for the above basic daily activities, who is helping you?**<br><br>**(Please tick all corresponding options)**	( )1. Spouse ( )2.Son ( )3.Daughter<br>( )4.Daughter-in-law ( )5. Son-in-law ( )6.Grandchild<br>( )7.Other relatives ( )8.Nurse ( )9.Friend<br>( )10.Neighbour ( )11.Community worker<br>( )12. Staff in Elderly Care Institution<br>( )13. Others (please specify) _______ ( )14. No one can help	
F3a. Continuing the question above, who is your main helper among these people?	The main helper__________ **(Please choose one from F3)**	☐
**F4. Are you able to take care of yourself in the following general daily activities?**		
a. Cooking	1. I can take care of myself without help<br>2. I can take care of myself, but need some help<br>3. I am not able to take care of myself	☐
b. Laundry	1. I can take care of myself without help<br>2. I can take care of myself, but need some help<br>3. I am not able to take care of myself	☐
c. Cleaning	1. I can take care of myself without help<br>2. I can take care of myself, but need some help<br>3. I am not able to take care of myself	☐
d. Taking medicine	1. I can take care of myself without help<br>2. I can take care of myself, but need some help<br>3. I am not able to take care of myself	☐
f. Managing money	1. I can take care of myself without help<br>2. I can take care of myself, but need some help<br>3. I am not able to take care of myself	☐

g. Making a call	1. I can take care of myself without help<br>2. I can take care of myself, but need some help<br>3. I am not able to take care of myself	☐
h. Taking public transports	1. I can take care of myself without help<br>2. I can take care of myself, but need some help<br>3. I am not able to take care of myself	☐
i. Shopping	1. I can take care of myself without help<br>2. I can take care of myself, but need some help<br>3. I am not able to take care of myself	☐
j. Seeing a doctor	1. I can take care of myself without help<br>2. I can take care of myself, but need some help<br>3. I am not able to take care of myself	☐
**F5. If you need help for the above general daily activities, who is helping you?**<br><br>**(Please tick all corresponding options)**	( )1. Spouse ( )2.Son ( )3.Daughter<br>( )4.Daughter-in-law ( )5. Son-in-law ( )6.Grandchild<br>( )7.other relatives ( )8.Nurse ( )9.Friend<br>( )10.Neighbour ( )11.Community worker<br>( )12. Staff in Elderly Care Institution<br>( )13. Others (please specify) _______ ( )14. No one can help	
F5a. Continuing the question above, who is your main helper among these people?	The main helper__________ **(Please choose one from F5)**	☐
**F6. How much can you afford per month, if you would like to live in Elderly Care Institution for your old age?**	(1)below 400 yuan<br>(2)400-599 yuan<br>(3)600-799yuan<br>(4)800-999 yuan<br>(5)1000-1199 yuan<br>(6)1200-1499 yuan<br>(7)1500-1999 yuan<br>(8)2000-2999 yuan<br>(9)3000 yuan and above	☐
**F7. Can you accept the idea of reversed mortgage?**	1. Yes 2. No 3. I have no idea	☐

G. Community and society					Code
**G1. Are there any of the following activity venues near your home? Have you used these venues?**<br><br>**Attendance level**<br>**1. Yes, and have used**<br>**2. Yes, but have not used（fill in 0）**<br>**3. No（fill in 0）**	**Types of venues**	**whether or not used it and how frequent**			
	a. Community service centre	1	2	3	☐
	b. Community service centre	1	2	3	☐
	c. Community culture centre	1	2	3	☐
	d. School for elderly	1	2	3	☐
	e. Gym or stadium	1	2	3	☐
	f. Day care/ aged care centre	1	2	3	☐
	g. Library	1	2	3	☐

**G2. Do you think you can find the following people in your life?**<br>1. Never 2. Rarely 3. Sometimes 4. Most of the time 5. Always						
a. someone you share happiness with	1.	2.	3.	4.	5.	☐
b. someone who cares about you	1.	2.	3.	4.	5.	☐
c. someone who plays with you	1.	2.	3.	4.	5.	☐
d. someone who can listen to you	1.	2.	3.	4.	5.	☐
e. someone who can give you hug	1.	2.	3.	4.	5.	☐
f. someone who solves your problem	1.	2.	3.	4.	5.	☐
g. someone who offers ideas and advice	1.	2.	3.	4.	5.	☐
h. someone who cooks for you when you are unable to cook	1.	2.	3.	4.	5.	☐
i. someone who can do housework for you when you are sick	1.	2.	3.	4.	5.	☐
j. someone who looks after you when you are sick in bed	1.	2.	3.	4.	5.	☐
k. someone who loves and protects you	1.	2.	3.	4.	5.	☐
l. someone you can relax with	1.	2.	3.	4.	5.	☐

**G3. How many family members or friends do you have who can listen to you in your life?**	______	☐☐
**G4. For the past half a year, how many people have you discussed important things with?**	______	☐☐

G5. G5. For the people you have discussed important things with, please briefly introduce the basic information of five of them:

a. relationships with you: 1. Family 2. Relatives 3. Friends 4. Colleagues 5. Acquaintance

b. gender： 1.Male 2.Female

c. age: ______years old

d. closeness： 1. Very close 2. Close 3. Not close 4. Not close at all

e. education level： 1. Below primary school 2. Primary school
3. Middle school 4. High school
5. Technical secondary school/Polytechnics/ Vocational school
6. Diploma 7.Bachelor
8. Master 9. PhD
10.Others（Please specify）____________

f. if this person suggests you to do medical examination, would you accept it? 1.Yes 2.No

	**a. Relationship with you**	**B .Gender**	**c. Age**	**d. Closeness**	**e. Education level**	**f. Advice on medical examination**	
No.1	a1. ☐	b1. ☐	c1. ☐☐	d1. ☐	e1. ☐☐	f1. ☐	
No.2	a2. ☐	b2. ☐	c2. ☐☐	d2. ☐	e2. ☐☐	f2. ☐	
No.3	a3. ☐	b3. ☐	c3. ☐☐	d3. ☐	e3. ☐☐	f3. ☐	
No.4	a4. ☐	b4. ☐	c4. ☐☐	d4. ☐	e4. ☐☐	f4. ☐	
No.5	a5. ☐	b5. ☐	c5. ☐☐	d5. ☐	e5. ☐☐	f5. ☐	

G6. The following is some specific occupations. For every specific occupation, please tell us if there is any people you know who is working in these professions. f you have a few people in mind, please use the first person you have in mind as an example.

a. Whether you know such a person: 1.Yes 2.No（**please skip to the next occupation**）

b. Relationship with you: 1.Family 2.Relative 3.Friend 4.Colleague 5. Acquaintance

c. Gender： 1.Male 2.Female

d. Age: ______years old

e. Closeness： 1. very close 2. close 3. not close 4. not close at all

f. Education level： 1. Below primary school 2. Primary school 3. Middle school
4. High school 5. Technical secondary school/Polytechnics/ Vocational school
6. Diploma 7.Bachelor 8.Master 9. PhD
10.Others（Please specify）____________

g. If this person suggests you do medical examination, would you accept it? 1.Yes. 2.No

	**a. Whether you know such a person**	**b. Relationship with you**	**c. Gender**	**d. Age**	**e. Closeness**	**f. Education level**	**g. Advice on medical examination**
1.Secondary school teacher	a1. ☐	b1. ☐	c1. ☐	d1. ☐☐	e1. ☐	h1. ☐☐	g1. ☐
2.Corporation manager	a2. ☐	b2. ☐	c2. ☐	d2. ☐☐	e2. ☐	h2. ☐☐	g2. ☐
3.Software developer	a3. ☐	b3. ☐	c3. ☐	d3. ☐☐	e3. ☐	h3. ☐☐	g3. ☐
4. Pharmacist	a4. ☐	b4. ☐	c4. ☐	d4. ☐☐	e4. ☐	h4. ☐☐	g4. ☐
5. Hairdresser	a5. ☐	b5. ☐	c5. ☐	d5. ☐☐	e5. ☐	h5. ☐☐	g5. ☐
6. Nurse	a6. ☐	b6. ☐	c6. ☐	d6. ☐☐	e6. ☐	h6. ☐☐	g6. ☐
7. Receptionist	a7. ☐	b7. ☐	c7. ☐	d7. ☐☐	e7. ☐	h7. ☐☐	g7. ☐
8. Doctor	a8. ☐	b8. ☐	c8. ☐	d8. ☐☐	e8. ☐	h8. ☐☐	g8. ☐
9. Cleaning staff	a9. ☐	b9. ☐	c9. ☐	d9. ☐☐	e9. ☐	h9. ☐☐	g9. ☐
10.Factory operator	a10. ☐	b10. ☐	c10. ☐	d10. ☐☐	e10. ☐	h10. ☐☐	g10. ☐

**G7. Which of the following views do you agree more?**	a. Most people are trustworthy<br>b. Be cautious when interact with other people	☐

G8. Are you a member of the following social groups?		
a. Religious group	1. Actively participant 2. Participate 3. not participate	☐
b. Exercise or recreational group	1. Actively participant 2. Participate 3. not participate	☐
c. Arts or educational group	1. Actively participant 2. Participate 3. not participate	☐
d. Union	1. Actively participant 2. Participate 3. not participate	☐
e. Political party	1. Actively participant 2. Participate 3. not participate	☐
f. Environmental protection organization	1. Actively participant 2. Participate 3. not participate	☐
g. Professional training clubs	1. Actively participant 2. Participate 3. not participate	☐
h. Humanitarian or charitable organization	1. Actively participant 2. Participate 3. not participate	☐
i. Self-help or mutual aid organization	1. Actively participant 2. Participate 3. not participate	☐
j. Others(please specify)________	1. Actively participant 2. Participate 3. not participate	☐
**G9. Are you volunteering currently?**	1.Yes 2.No **(please skip to G10)**	☐
G9a. How long do you spend on volunteering weekly last month on average?	1. less than 5 hours 2. 6—10 hours 3. 11—15 hours<br>4. 16—20 hours 5. 21—25 hours 6. 26—30 hours<br>7. more than 30 hours	☐

**G10. What are the things you support your children with currently?**<br><br>**Support level:**<br>**0.No support**<br>**1.Little support**<br>**2.A lot of support**<br>**8.Not applicable**	**Support items**	**Support level**				
		**No support**	**Little support**	**A lot of support**	**Not application**	
	1. Financial support	0	1	2	8	☐
	2. Career support	0	1	2	8	☐
	3.Household support	0	1	2	8	☐
	4. Looking after grandchildren	0	1	2	8	☐
	5.others:________	0	1	2	8	☐

H. Overall rating			Code
**H1. To achieve a "successful lifestyle of the elderly", how do you think the importance of the following?**			
a. Independent living	1.Very important 2. Important 3. Somewhat important 4. Not		☐
b. Having friends	1.Very important 2. Important 3. Somewhat important 4. Not		☐
c. Living with children	1.Very important 2. Important 3. Somewhat important 4. Not		☐
d. Good financial status	1.Very important 2. Important 3. Somewhat important 4. Not		☐
e. Good health	1.Very important 2. Important 3. Somewhat important 4. Not		☐
f. No diseases	1.Very important 2. Important 3. Somewhat important 4. Not		☐
g. Being able continue working	1.Very important 2. Important 3. Somewhat important 4. Not		☐
h. Being looked after by children	1.Very important 2. Important 3. Somewhat important 4. Not		☐
i. Attending social events	1.Very important 2. Important 3. Somewhat important 4. Not		☐
j. Having partners around	1.Very important 2. Important 3. Somewhat important 4. Not		☐
k. Being able to take care of family	1.Very important 2. Important 3. Somewhat important 4. Not		☐
l. Feeling happy	1.Very important 2. Important 3. Somewhat important 4. Not		☐
**H2. According to your views on "successful lifestyle of the elderly", are you enjoying the "successful lifestyle of the elderly" now?**		1 Yes 2. No	☐
**H3. Are you satisfied with the following aspects of your life?**			
a. Your health condition	1. Very satisfied 2. Satisfied 3. Unsatisfied 4. Very unsatisfied 5. Not sure		☐
b. Your financial status	1. Very satisfied 2. Satisfied 3. Unsatisfied 4. Very unsatisfied 5. Not sure		☐
c. can have peace of mind	1. Very satisfied 2. Satisfied 3. Unsatisfied 4. Very unsatisfied 5. Not sure		☐
d. Your social life	1. Very satisfied 2. Satisfied 3. Unsatisfied 4. Very unsatisfied 5. Not sure		☐
e. Your family relationship	1. Very satisfied 2. Satisfied 3. Unsatisfied 4. Very unsatisfied 5. Not sure		☐
f. Your marital status	1. Very satisfied 2. Satisfied 3. Unsatisfied 4. Very unsatisfied 5. Not sure		☐
g. Financial support of children	1. Very satisfied 2. Satisfied 3. Unsatisfied 4. Very unsatisfied 5. Not sure		☐
h. Daily care from children	1. Very satisfied 2. Satisfied 3. Unsatisfied 4. Very unsatisfied 5. Not sure		☐
i. Emotional support of children	1. Very satisfied 2. Satisfied 3. Unsatisfied 4. Very unsatisfied 5. Not sure		☐
j. Your living situation	1. Very satisfied 2. Satisfied 3. Unsatisfied 4. Very unsatisfied 5. Not sure		☐
l. Your work situation	1. Very satisfied 2. Satisfied 3. Unsatisfied 4. Very unsatisfied 5. Not sure		☐
n. The level of respectfulness	1. Very satisfied 2. Satisfied 3. Unsatisfied 4. Very unsatisfied 5. Not sure		☐
s. Your overall life	1. Very satisfied 2. Satisfied 3. Unsatisfied 4. Very unsatisfied 5. Not sure		☐

This is the end of this questionnaire. Thanks very much your help and support!

**Interviewers need to fill the following questions**		**Code**
I. **Are there any other people in the scene during the process of investigation?**	1.Yes, most of the time in the scene<br>2. Yes, approximately half of the time in the scene<br>3. Yes, occasionally in the scene<br>4. No, no one in the scene **(until the completion of the questionnaire)**	□
a. What are the relationships between the elderly participant and the people in the scene?<br>(multiple choice, please tick the corresponding items)	( )1. Spouse ( )2. Son<br>( )3. Daughter ( )4. Daughter-in-law<br>( )5.Son-in-law ( )6.Grandchildren<br>( )7.other relatives ( )8.Nurse<br>( )9.Neighbour ( )10.government officials<br>( )11.others (please specify) ______________	□□□<br>□□□<br>□□□<br>□□
b. Have the other people in the scene answered any questions for the elderly participant?	1. Yes 2. No	□

Interviewers need to double check for any missed questions now.

If there is no missing item, please tick here____________.
