# Supplementary material for: Belief in and use of traditional Chinese medicine in Shanghai older adults: a cross-sectional study
Source: BMC Complement Med Ther. 2020 Apr 28;20:128. doi: 10.1186/s12906-020-02910-x (PMC7189641; doi:10.1186/s12906-020-02910-x)
Supplement: Supplementary file 2 — Additional file 2. Supplementary materials. [file 12906_2020_2910_MOESM2_ESM.docx]

**Supplementary materials**

Table 3A: Crude Odds Ratios and 95% Confidence Intervals for Variables in Table 3

|  | **Crude OR** | **95% CI** |
| --- | --- | --- |
| ***Demographics*** |  |  |
| Gender (male=1, female=0) | 0.75 | [0.66, 0.86] |
| Age (range: 50-100) | 1.01 | [1.00, 1.02] |
| Residence (urban=1, rural=0) | 0.70 | [0.54, 0.91] |
|  |  |  |
| ***Medical factors*** |  |  |
| Hypertension (yes=1, no=0) | 1.22 | [1.07, 1.40] |
| Cardiovascular diseases (yes=1, no=0) | 1.63 | [1.38, 1.92] |
| Stroke (yes=1, no=0) | 1.15 | [0.86, 1.54] |
| Pneumonia/…/ asthma (yes=1, no=0) | 1.69 | [1.37, 2.10] |
| Diabetes (yes=1, no=0) | 1.23 | [0.99, 1.53] |
| Depression (yes=1, no=0) | 1.66 | [0.86, 3.17] |
| Cancer (yes=1, no=0) | 3.47 | [2.11, 5.71] |
| Prostatitis (yes=1, no=0) | 1.67 | [1.22, 2.29] |
| Osteoporosis/osteoarthritis (yes=1, no=0) | 1.47 | [1.24, 1.74] |
| Fracture (yes=1, no=0) | 1.73 | [1.24, 2.41] |
| Parkinson’s disease (yes=1, no=0) | 0.77 | [0.33, 1.80] |
| Nervous system disease (yes=1, no=0) | 1.63 | [1.19, 2.24] |
|  |  |  |
| ***Socioeconomics*** |  |  |
| Education (primary school or below=0) |  |  |
| Middle school | 0.93 | [0.77, 1.11] |
| High/polytechnic school | 1.03 | [0.85, 1.25] |
| Colleague or above. | 1.43 | [1.13, 1.82] |
| Monthly income (range: 0-10,000 yuan) | 1.03 | [0.97, 1.09] |
| Main occupation (professional/administrative=0) |  |  |
| Clerk/service | 0.64 | [0.52, 0.80] |
| Physical labor | 0.73 | [0.61, 0.88] |
| Other | 0.75 | [0.58, 0.96] |
|  |  |  |
| ***Relational and contextual factors*** |  |  |
| Spouse accompany (yes=1, no=0) | 1.01 | [0.86, 1.19] |
| Number of strong ties to confide | 1.05 | [1.03, 1.07] |
| Knowing medical staff (yes=1, no=0) | 1.26 | [1.05, 1.51] |
| Number of community facilities | 1.03 | [1.00, 1.06] |
| GDP per capita in the district (unit: 10,000 yuan) | 1.05 | [0.91, 1.21] |
| Medical staff per 10,000 residents in the district | 1.01 | [0.98, 1.05] |

Table 3B: Odds Ratios and 95% Confidence Intervals for Model I in Table 3

|  | **OR** | **95% CI** |
| --- | --- | --- |
| ***Demographics*** |  |  |
| Gender (male=1, female=0) | 0.76 | [0.66, 0.87] |
| Age (range: 50-100) | 1.01 | [1.00, 1.01] |
| Residence (urban=1, rural=0) | 0.71 | [0.55, 0.93] |
|  |  |  |
| Intercept | 0.78 | [0.44, 1.40] |

Table 3C: Odds Ratios and 95% Confidence Intervals for Model II in Table 3

|  | **OR** | **95% CI** | |
| --- | --- | --- | --- |
| ***Demographics*** |  |  | |
| Gender (male=1, female=0) | 0.75 | [0.65, 0.87] | |
| Age (range: 50-100) | 1.00 | [0.99, 1.01] | |
| Residence (urban=1, rural=0) | 0.72 | [0.55, 0.94] | |
|  |  |  | |
| ***Medical factors*** |  |  | |
| Hypertension (yes=1, no=0) | 1.17 | [1.01, 1.35] | |
| Cardiovascular diseases (yes=1, no=0) | 1.45 | [1.22, 1.72] | |
| Stroke (yes=1, no=0) | 0.95 | [0.70, 1.29] | |
| Pneumonia/…/ asthma (yes=1, no=0) | 1.53 | [1.22, 1.90] | |
| Diabetes (yes=1, no=0) | 1.16 | [0.92, 1.45] | |
| Depression (yes=1, no=0) | 1.45 | [0.73, 2.89] | |
| Cancer (yes=1, no=0) | 3.64 | [2.20, 6.03] | |
| Prostatitis (yes=1, no=0) | 1.71 | [1.22, 2.39] | |
| Osteoporosis/osteoarthritis (yes=1, no=0) | 1.30 | [1.09, 1.55] | |
| Fracture (yes=1, no=0) | 1.45 | [1.03, 2.05] | |
| Parkinson’s disease (yes=1, no=0) | 0.56 | [0.23, 1.36] | |
| Nervous system disease (yes=1, no=0) | 1.39 | [1.00, 1.94] | |
|  |  |  |  |
| Intercept | 1.00 | [0.55, 1.82] | |

Table 3D: Odds Ratios and 95% Confidence Intervals for Model III in Table 3

|  | **OR** | **95% CI** |
| --- | --- | --- |
| ***Demographics*** |  |  |
| Gender (male=1, female=0) | 0.69 | [0.60, 0.80] |
| Age (range: 50-100) | 1.01 | [1.00, 1.02] |
| Residence (urban=1, rural=0) | 0.62 | [0.46, 0.83] |
|  |  |  |
| ***Socioeconomics*** |  |  |
| Education (primary school or below=0) |  |  |
| Middle school | 1.18 | [0.95, 1.45] |
| High/polytechnic school | 1.34 | [1.05, 1.71] |
| Colleague or above. | 1.72 | [1.27, 2.35] |
| Monthly income (range: 0-10,000 yuan) | 1.02 | [0.96, 1.09] |
| Main occupation (professional/administrative=0) |  |  |
| Clerk/service | 0.74 | [0.59, 0.94] |
| Physical labor | 0.85 | [0.68, 1.07] |
| Other | 0.83 | [0.61, 1.12] |
|  |  |  |
| Intercept | 0.71 | [0.34, 1.48] |

Table 3E: Odds Ratios and 95% Confidence Intervals for Model IV in Table 3

|  | **OR** | **95% CI** |
| --- | --- | --- |
| ***Demographics*** |  |  |
| Gender (male=1, female=0) | 0.76 | [0.65, 0.87] |
| Age (range: 50-100) | 1.01 | [1.00, 1.02] |
| Residence (urban=1, rural=0) | 0.76 | [0.58, 1.00] |
|  |  |  |
| ***Relational and contextual factors*** |  |  |
| Spouse accompany (yes=1, no=0) | 1.14 | [0.95, 1.37] |
| Number of strong ties to confide | 1.05 | [1.03, 1.07] |
| Knowing medical staff (yes=1, no=0) | 1.19 | [0.99, 1.44] |
| Number of community facilities | 1.03 | [0.99, 1.06] |
| GDP per capita in the district (unit: 10,000 yuan) | 1.00 | [0.81, 1.23] |
| Medical staff per 10,000 residents in the district | 1.02 | [0.97, 1.08] |
|  |  |  |
| Intercept | 0.27 | [0.12, 0.59] |

Table 3F: Odds Ratios and 95% Confidence Intervals for Model V in Table 3

|  | **OR** | **95% CI** |
| --- | --- | --- |
| ***Demographics*** |  |  |
| Gender (male=1, female=0) | 0.68 | [0.58, 0.80] |
| Age (range: 50-100) | 1.01 | [1.00, 1.01] |
| Residence (urban=1, rural=0) | 0.68 | [0.50, 0.93] |
|  |  |  |
| ***Medical factors*** |  |  |
| Hypertension (yes=1, no=0) | 1.14 | [0.98, 1.32] |
| Cardiovascular diseases (yes=1, no=0) | 1.45 | [1.21, 1.72] |
| Stroke (yes=1, no=0) | 0.99 | [0.73, 1.35] |
| Pneumonia/…/ asthma (yes=1, no=0) | 1.56 | [1.25, 1.95] |
| Diabetes (yes=1, no=0) | 1.19 | [0.95, 1.49] |
| Depression (yes=1, no=0) | 1.54 | [0.77, 3.10] |
| Cancer (yes=1, no=0) | 3.39 | [2.03, 5.65] |
| Prostatitis (yes=1, no=0) | 1.61 | [1.14, 2.25] |
| Osteoporosis/osteoarthritis (yes=1, no=0) | 1.32 | [1.10, 1.58] |
| Fracture (yes=1, no=0) | 1.40 | [0.98, 1.99] |
| Parkinson’s disease (yes=1, no=0) | 0.57 | [0.23, 1.41] |
| Nervous system disease (yes=1, no=0) | 1.45 | [1.03, 2.02] |
|  |  |  |
| ***Socioeconomics*** |  |  |
| Education (primary school or below=0) |  |  |
| Middle school | 1.21 | [0.97, 1.50] |
| High/polytechnic school | 1.38 | [1.07, 1.78] |
| Colleague or above. | 1.75 | [1.28, 2.41] |
| Monthly income (range: 0-10,000 yuan) | 1.02 | [0.95, 1.09] |
| Main occupation (professional/administrative=0) |  |  |
| Clerk/service | 0.78 | [0.61, 0.99] |
| Physical labor | 0.90 | [0.72, 1.13] |
| Other | 0.91 | [0.67, 1.24] |
|  |  |  |
| ***Relational and contextual factors*** |  |  |
| Spouse accompany (yes=1, no=0) | 1.13 | [0.93, 1.37] |
| Number of strong ties to confide | 1.04 | [1.02, 1.07] |
| Knowing medical staff (yes=1, no=0) | 1.08 | [0.89, 1.32] |
| Number of community facilities | 1.02 | [0.99, 1.06] |
| GDP per capita in the district (unit: 10,000 yuan) | 1.05 | [0.86, 1.28] |
| Medical staff per 10,000 residents in the district | 1.01 | [0.96, 1.06] |
|  |  |  |
| Intercept | 0.33 | [0.13, 0.83] |

Table 4A: Crude Odds Ratios and 95% Confidence Intervals for Variables in Table 4

|  | **Crude OR** | **95% CI** |
| --- | --- | --- |
| ***Demographics*** |  |  |
| Gender (male=1, female=0) | 0.91 | [0.79, 1.04] |
| Age (range: 50-100) | 0.99 | [0.99, 1.00] |
| Residence (urban=1, rural=0) | 0.76 | [0.58, 0.99] |
|  |  |  |
| ***Medical factors*** |  |  |
| Hypertension (yes=1, no=0) | 0.97 | [0.84, 1.11] |
| Cardiovascular diseases (yes=1, no=0) | 1.09 | [0.93, 1.29] |
| Stroke (yes=1, no=0) | 0.80 | [0.60, 1.08] |
| Pneumonia/…/ asthma (yes=1, no=0) | 1.00 | [0.81, 1.24] |
| Diabetes (yes=1, no=0) | 0.95 | [0.76, 1.18] |
| Depression (yes=1, no=0) | 1.12 | [0.58, 2.18] |
| Cancer (yes=1, no=0) | 2.16 | [1.35, 3.44] |
| Prostatitis (yes=1, no=0) | 1.54 | [1.12, 2.12] |
| Osteoporosis/osteoarthritis (yes=1, no=0) | 0.88 | [0.74, 1.05] |
| Fracture (yes=1, no=0) | 1.39 | [1.00, 1.94] |
| Parkinson’s disease (yes=1, no=0) | 0.53 | [0.21, 1.32] |
| Nervous system disease (yes=1, no=0) | 0.78 | [0.56, 1.07] |
|  |  |  |
| ***Socioeconomics*** |  |  |
| Education (primary school or below=0) |  |  |
| Middle school | 1.19 | [0.99, 1.43] |
| High/polytechnic school | 1.56 | [1.28, 1.90] |
| Colleague or above. | 1.98 | [1.55, 2.53] |
| Monthly income (range: 0-10,000 yuan) | 1.11 | [1.05, 1.18] |
| Main occupation (professional/administrative=0) |  |  |
| Clerk/service | 0.69 | [0.55, 0.85] |
| Physical labor | 0.59 | [0.49, 0.71] |
| Other | 0.57 | [0.44, 0.73] |
|  |  |  |
| ***Relational and contextual factors*** |  |  |
| Spouse accompany (yes=1, no=0) | 1.26 | [1.06, 1.49] |
| Number of strong ties to confide | 1.07 | [1.05, 1.09] |
| Knowing medical staff (yes=1, no=0) | 1.67 | [1.39, 2.02] |
| Number of community facilities | 1.03 | [1.00, 1.07] |
| GDP per capita in the district (unit: 10,000 yuan) | 1.00 | [0.83, 1.19] |
| Medical staff per 10,000 residents in the district | 1.00 | [0.96, 1.05] |

Table 4B: Odds Ratios and 95% Confidence Intervals for Model I in Table 4

|  | **OR** | **95% CI** |
| --- | --- | --- |
| ***Demographics*** |  |  |
| Gender (male=1, female=0) | 0.90 | [0.78, 1.03] |
| Age (range: 50-100) | 0.99 | [0.98, 1.00] |
| Residence (urban=1, rural=0) | 0.74 | [0.56, 0.96] |
|  |  |  |
| Intercept | 2.18 | [1.19, 4.01] |

Table 4C: Odds Ratios and 95% Confidence Intervals for Model II in Table 4

|  | **OR** | **95% CI** |
| --- | --- | --- |
| ***Demographics*** |  |  |
| Gender (male=1, female=0) | 0.84 | [0.73, 0.97] |
| Age (range: 50-100) | 0.99 | [0.98, 1.00] |
| Residence (urban=1, rural=0) | 0.69 | [0.52, 0.90] |
|  |  |  |
| ***Medical factors*** |  |  |
| Hypertension (yes=1, no=0) | 0.98 | [0.85, 1.14] |
| Cardiovascular diseases (yes=1, no=0) | 1.16 | [0.97, 1.38] |
| Stroke (yes=1, no=0) | 0.84 | [0.62, 1.14] |
| Pneumonia/…/ asthma (yes=1, no=0) | 1.02 | [0.82, 1.27] |
| Diabetes (yes=1, no=0) | 0.97 | [0.77, 1.21] |
| Depression (yes=1, no=0) | 1.25 | [0.63, 2.49] |
| Cancer (yes=1, no=0) | 2.10 | [1.31, 3.36] |
| Prostatitis (yes=1, no=0) | 1.74 | [1.25, 2.43] |
| Osteoporosis/osteoarthritis (yes=1, no=0) | 0.85 | [0.71, 1.01] |
| Fracture (yes=1, no=0) | 1.51 | [1.08, 2.12] |
| Parkinson’s disease (yes=1, no=0) | 0.55 | [0.22, 1.39] |
| Nervous system disease (yes=1, no=0) | 0.71 | [0.51, 0.99] |
|  |  |  |
| Intercept | 2.57 | [1.37, 4.79] |

Table 4D: Odds Ratios and 95% Confidence Intervals for Model III in Table 4

|  | **OR** | **95% CI** |
| --- | --- | --- |
| ***Demographics*** |  |  |
| Gender (male=1, female=0) | 0.79 | [0.68, 0.92] |
| Age (range: 50-100) | 1.00 | [0.99, 1.00] |
| Residence (urban=1, rural=0) | 0.53 | [0.39, 0.71] |
|  |  |  |
| ***Socioeconomics*** |  |  |
| Education (primary school or below=0) |  |  |
| Middle school | 1.21 | [0.98, 1.50] |
| High/polytechnic school | 1.50 | [1.17, 1.92] |
| Colleague or above. | 1.70 | [1.25, 2.33] |
| Monthly income (range: 0-10,000 yuan) | 1.07 | [1.00, 1.14] |
| Main occupation (professional/administrative=0) |  |  |
| Clerk/service | 0.77 | [0.61, 0.98] |
| Physical labor | 0.73 | [0.58, 0.91] |
| Other | 0.65 | [0.48, 0.88] |
|  |  |  |
| Intercept | 2.02 | [0.94, 4.33] |

Table 4E: Odds Ratios and 95% Confidence Intervals for Model IV in Table 4

|  | **OR** | **95% CI** |
| --- | --- | --- |
| ***Demographics*** |  |  |
| Gender (male=1, female=0) | 0.89 | [0.77, 1.02] |
| Age (range: 50-100) | 1.00 | [0.99, 1.00] |
| Residence (urban=1, rural=0) | 0.80 | [0.61, 1.06] |
|  |  |  |
| ***Relational and contextual factors*** |  |  |
| Spouse accompany (yes=1, no=0) | 1.16 | [0.96, 1.40] |
| Number of strong ties to confide | 1.05 | [1.03, 1.08] |
| Knowing medical staff (yes=1, no=0) | 1.54 | [1.27, 1.87] |
| Number of community facilities | 1.02 | [0.99, 1.06] |
| GDP per capita in the district (unit: 10,000 yuan) | 0.98 | [0.74, 1.30] |
| Medical staff per 10,000 residents in the district | 1.02 | [0.95, 1.10] |
|  |  |  |
| Intercept | 0.70 | [0.28, 1.73] |

Table 4F: Odds Ratios and 95% Confidence Intervals for Model V in Table 4

|  | **OR** | **95% CI** |
| --- | --- | --- |
| ***Demographics*** |  |  |
| Gender (male=1, female=0) | 0.76 | [0.65, 0.89] |
| Age (range: 50-100) | 1.00 | [0.99, 1.01] |
| Residence (urban=1, rural=0) | 0.58 | [0.43, 0.79] |
|  |  |  |
| ***Medical factors*** |  |  |
| Hypertension (yes=1, no=0) | 0.95 | [0.82, 1.10] |
| Cardiovascular diseases (yes=1, no=0) | 1.14 | [0.96, 1.37] |
| Stroke (yes=1, no=0) | 0.89 | [0.65, 1.21] |
| Pneumonia/…/ asthma (yes=1, no=0) | 1.04 | [0.83, 1.30] |
| Diabetes (yes=1, no=0) | 1.01 | [0.80, 1.26] |
| Depression (yes=1, no=0) | 1.37 | [0.68, 2.77] |
| Cancer (yes=1, no=0) | 1.82 | [1.13, 2.94] |
| Prostatitis (yes=1, no=0) | 1.63 | [1.16, 2.29] |
| Osteoporosis/osteoarthritis (yes=1, no=0) | 0.85 | [0.71, 1.02] |
| Fracture (yes=1, no=0) | 1.44 | [1.02, 2.04] |
| Parkinson’s disease (yes=1, no=0) | 0.56 | [0.22, 1.43] |
| Nervous system disease (yes=1, no=0) | 0.74 | [0.53, 1.04] |
|  |  |  |
| ***Socioeconomics*** |  |  |
| Education (primary school or below=0) |  |  |
| Middle school | 1.23 | [0.98, 1.53] |
| High/polytechnic school | 1.49 | [1.15, 1.91] |
| Colleague or above. | 1.62 | [1.18, 2.22] |
| Monthly income (range: 0-10,000 yuan) | 1.05 | [0.98, 1.13] |
| Main occupation (professional/administrative=0) |  |  |
| Clerk/service | 0.82 | [0.64, 1.04] |
| Physical labor | 0.77 | [0.61, 0.97] |
| Other | 0.73 | [0.54, 1.00] |
|  |  |  |
| ***Relational and contextual factors*** |  |  |
| Spouse accompany (yes=1, no=0) | 1.08 | [0.89, 1.30] |
| Number of strong ties to confide | 1.05 | [1.03, 1.07 |
| Knowing medical staff (yes=1, no=0) | 1.42 | [1.17, 1.73] |
| Number of community facilities | 1.02 | [0.98, 1.05] |
| GDP per capita in the district (unit: 10,000 yuan) | 1.03 | [0.79, 1.35] |
| Medical staff per 10,000 residents in the district | 1.00 | [0.93, 1.08] |
|  |  |  |
| Intercept | 0.91 | [0.33, 2.52] |

Table A: Odds Ratios of Correlatives for Use and Belief of Traditional Medicine Based on Multi-level Logit Regression Models, with the Variable of Number of Medical Conditions

|  | **Use** | **Belief** |
| --- | --- | --- |
| ***Demographics*** |  |  |
| Gender (male=1, female=0) | 0.681*** | 0.758*** |
| Age (range: 50-100) | 1.005 | 0.999 |
| Residence (urban=1, rural=0) | 0.682* | 0.579*** |
|  |  |  |
| ***Medical factors*** |  |  |
| Hypertension (yes=1, no=0) | 0.957 | 0.944 |
| Cardiovascular diseases (yes=1, no=0) | 1.216 | 1.136 |
| Stroke (yes=1, no=0) | 0.835 | 0.881 |
| Pneumonia/…/ asthma (yes=1, no=0) | 1.312 | 1.035 |
| Diabetes (yes=1, no=0) | - | - |
| Depression (yes=1, no=0) | 1.297 | 1.364 |
| Cancer (yes=1, no=0) | 2.855*** | 1.813* |
| Prostatitis (yes=1, no=0) | 1.351 | 1.617* |
| Osteoporosis/osteoarthritis (yes=1, no=0) | 1.109 | 0.843 |
| Fracture (yes=1, no=0) | 1.177 | 1.431 |
| Parkinson’s disease (yes=1, no=0) | 0.482 | 0.552 |
| Nervous system disease (yes=1, no=0) | 1.217 | 0.739 |
| Number of Medical Conditions | 1.188* | 1.006 |
|  |  |  |
| ***Socioeconomics*** |  |  |
| Education (primary school or below=0) |  |  |
| Middle school | 1.206 | 1.225 |
| High/polytechnic school | 1.382* | 1.485** |
| Colleague or above. | 1.753*** | 1.618** |
| Monthly income (range: 0-10,000 yuan) | 1.017 | 1.053 |
| Main occupation (professional/administrative=0) |  |  |
| Clerk/service | 0.778* | 0.820 |
| Physical labor | 0.902 | 0.767* |
| Other | 0.910 | 0.733 |
|  |  |  |
| ***Relational and contextual factors*** |  |  |
| Spouse accompany (yes=1, no=0) | 1.129 | 1.078 |
| Number of strong ties to confide | 1.044*** | 1.052*** |
| Knowing medical staff (yes=1, no=0) | 1.085 | 1.420*** |
| Number of community facilities | 1.022 | 1.015 |
| GDP per capita in the district (unit: 10,000 yuan) | 1.049 | 1.031 |
| Medical staff per 10,000 residents in the district | 1.008 | 1.002 |
|  |  |  |
| Intercept | 0.329 | 0.911 |
|  |  |  |
| Log Likelihood | -2217.10 | -2215.69 |
| Variance of District Level Intercept | 0.12 | 0.22 |
| Sample size | 3408 | 3408 |

*** p < 0.001, ** p < 0.01, * p < 0.05

Table B: Odds Ratios of Correlatives for Use and Belief of Traditional Medicine Based on Multi-level Logit Regression Models, with the Variable of Types of Disease and Condition

|  | **Use** | **Belief** |
| --- | --- | --- |
| ***Demographics*** |  |  |
| Gender (male=1, female=0) | 0.688*** | 0.789** |
| Age (range: 50-100) | 1.005 | 0.999 |
| Residence (urban=1, rural=0) | 0.706* | 0.611** |
|  |  |  |
| ***Types of Disease and Condition (no disease and condition=0)*** |  |  |
| Hypertension | 1.182 | 0.947 |
| Osteoporosis | 1.424* | 0.863 |
| Hypertension + Cardiovascular disease | 2.323*** | 1.201 |
| Cardiovascular disease | 1.481* | 1.210 |
| Hypertension + Osteoporosis | 1.958** | 0.900 |
| Hypertension + Diabetes | 1.698* | 1.142 |
| Pneumonia/…/ asthma | 2.310*** | 0.990 |
| Hypertension + Cardiovascular disease + Osteoporosis | 1.920** | 0.702 |
| Diabetes | 0.607 | 0.693 |
| Other disease/condition | 2.384*** | 1.123 |
|  |  |  |
| ***Socioeconomics*** |  |  |
| Education (primary school or below=0) |  |  |
| Middle school | 1.217 | 1.236 |
| High/polytechnic school | 1.378* | 1.513** |
| Colleague or above. | 1.747 | 1.671** |
| Monthly income (range: 0-10,000 yuan) | 1.025 | 1.057 |
| Main occupation (professional/administrative=0) |  |  |
| Clerk/service | 0.766* | 0.810 |
| Physical labor | 0.875 | 0.759* |
| Other | 0.871 | 0.710* |
|  |  |  |
| ***Relational and contextual factors*** |  |  |
| Spouse accompany (yes=1, no=0) | 1.124 | 1.084 |
| Number of strong ties to confide | 1.044*** | 1.054*** |
| Knowing medical staff (yes=1, no=0) | 1.112 | 1.426*** |
| Number of community facilities | 1.022 | 1.016 |
| GDP per capita in the district (unit: 10,000 yuan) | 1.052 | 1.031 |
| Medical staff per 10,000 residents in the district | 1.007 | 1.003 |
|  |  |  |
| Intercept | 0.314* | 0.812 |
|  |  |  |
| Log Likelihood | -2218 | -2225 |
| Variance of District Level Intercept | 0.12 | 0.23 |
| Sample size | 3408 | 3408 |

*** p < 0.001, ** p < 0.01, * p < 0.05
